# Supplementary material for: The interactome of intact mitochondria by cross-linking mass spectrometry provides evidence for coexisting respiratory supercomplexes
Source: Mol Cell Proteomics. 2017 Dec 8;17(2):216–32. doi: 10.1074/mcp.RA117.000470 (PMC5795388; doi:10.1074/mcp.RA117.000470)
Supplement: Supplemental Data [file supp_RA117.000470_133922_0_supp_23978_fzffwf.zip › spectra_annotation/mito_spectra_annotation/291-1-9-1-5-1.pdf]

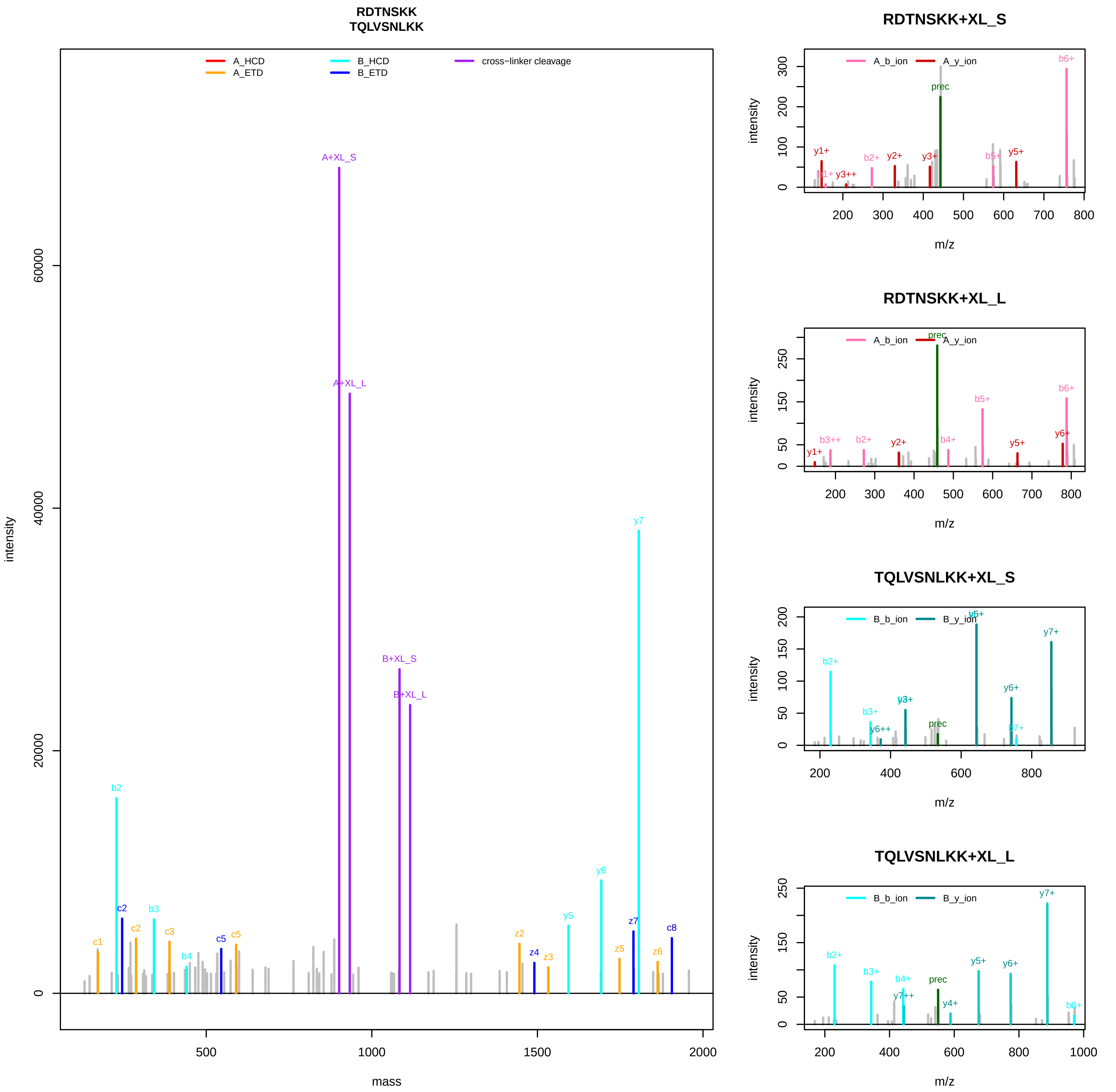
RDTNSKK
TQLVSNLKK
A_HCD
A_ETD
B_HCD
B_ETD
cross−linker cleavage
A+XL_S
A+XL_L
B+XL_S
B+XL_L
b2
b3
b4
c1
c2
c3
c5
c8
y5
y6
y7
z2
z3
z4
z5
z6
z7
intensity
mass
0
20000
40000
60000
500
1000
1500
2000
RDTNSKK+XL_S
A_b_ion
A_y_ion
b6+
prec
y1+
b1+
y3++
b2+
y2+
y3+
b5+
y5+
intensity
m/z
RDTNSKK+XL_L
b3++
b4+
y6+
TQLVSNLKK+XL_S
B_b_ion
B_y_ion
y5+
y7+
y6+
b3+
y6++
b7+
TQLVSNLKK+XL_L
y7++
y4+
b8+
